# Emerging Roles of Repetitive and Repeat-Containing RNA in Nuclear and Chromatin Organization and Gene Expression

**DOI:** 10.3389/fcell.2021.735527

**Published:** 2021-10-06

**Authors:** Giuseppe Trigiante, Nerea Blanes Ruiz, Andrea Cerase

**Affiliations:** Centre for Genomics and Child Health, Barts and The London School of Medicine and Dentistry, Blizard Institute, Queen Mary University of London, London, United Kingdom

**Keywords:** epigenetics, nuclear organization, long non-coding RNA, tandem repeats, repetitive RNA, membraneless compartments, X chromosome inactivation (XCI), *Xist (X-inactive specific transcript)*

## Abstract

Genomic repeats have been intensely studied as regulatory elements controlling gene transcription, splicing and genome architecture. Our understanding of the role of the repetitive RNA such as the RNA coming from genomic repeats, or repetitive sequences embedded in mRNA/lncRNAs, in nuclear and cellular functions is instead still limited. In this review we discuss evidence supporting the multifaceted roles of repetitive RNA and RNA binding proteins in nuclear organization, gene regulation, and in the formation of dynamic membrane-less aggregates. We hope that our review will further stimulate research in the consolidating field of repetitive RNA biology.

## Non-Coding RNAs in the Nucleus

The eukaryotic nucleus is a very complex organelle containing large amounts of DNA, RNA, proteins (Boija et al., 2018; Sawyer et al., 2019) and nuclear compartments, such as membrane-less organelles like the nucleoli, speckles, paraspeckles. These organelles are droplet-like particles that exhibit liquid-like features and are believed to be formed via a process called liquid-liquid phase separation (LLPS, discussed below).

The mechanisms behind the differential expression of protein coding and non-coding genes in time, space and in response to external stimuli, is the object of intensive studies. It is well known that, transcriptionally, chromatin exists in one of two main states: euchromatin, actively transcribed and not condensed, and heterochromatin, relatively compacted, gene-poor and transcriptionally silent (Trojer and Reinberg, 2007), and that these share different nuclear compartments (Van Bortle and Corces, 2012). The fraction of the heterochromatin that is permanently silent is called constitutive heterochromatin, whereas the fraction of chromatin that undergoes transcriptional activation or repression depending on cellular, extracellular and developmental clues is called facultative heterochromatin. Constitutive heterochromatin is enriched for repetitive DNA (for example the centromeric and telomeric regions and genomic repeats) (Nishibuchi and Déjardin, 2017).

While the role of repetitive genomic elements has been intensely studied, the role of repetitive or repeat-containing RNA has only relatively recently gained some attention. It is now becoming increasingly clear that mRNAs and nuclear non-coding RNAs are centrally involved in the regulation of chromatin state and therefore gene expression. For example, it has been known for decades that RNA can be intimately associated to chromatin (Holmes and Bonner, 1974). This type of RNA is now collectively labeled as chromatin-associated RNA (caRNA) (Li and Fu, 2019). This term encompasses a wide variety of specific RNAs such as short nuclear/nucleolar (snRNA/snoRNA) and long non-coding RNA (lncRNA) and newly transcribed mRNA (Li and Fu, 2019). While their specific action mechanism is mostly unknown, it is now generally believed that they act in combination with RNA-binding proteins (RBP) and histone modifying proteins such as, for example, the Suppressor of Variegation 3–9 (SUV39), Polycomb Repressor complex (PRC1 and 2) (Davidovich and Cech, 2015; Cerase and Tartaglia, 2020), and hNRNP-family protein (Klimek-Tomczak et al., 2004; West et al., 2019) to regulate gene expression and nuclear dynamics.

Due to recent excellent reviews on the topic of lncRNA and RNA in general nuclear organization (Creamer and Lawrence, 2017; Khosraviani et al., 2019; Michieletto and Gilbert, 2019; Thakur et al., 2019), this review will focus on the emerging evidence of the role of repetitive RNA and repeat-containing repetitive motifs (i.e., genomic, simple/tandem containing repeats) in the organization of chromatin and the regulation of gene expression.

## Repetitive caRNA

Repetitive caRNAs are transcribed from repetitive DNA sequences such as DNA-containing genomic repeats, fragments of genomic repeats or repeated motifs such as simple repeats (Biscotti et al., 2015). It is believed that repetitive sequences make up at least half of the human genome, with some estimates placing it at two thirds (de Koning et al., 2011). Such sequences were once labeled the “dark matter” of the genome or “junk DNA,” but it is becoming more and more clear that they instead play critical roles in regulating gene expression at different levels (Statello et al., 2021). They can be classified in two broad categories: low complexity, consisting of adjacent sequences repeated in tandem, and interspersed repeats, complex sequences generally capable of being transcribed and sometimes translated. Examples of tandem repeats are the telomeric and centromeric repeats and satellite DNA (Janssen et al., 2018). These repeats are often associated with a constitutive heterochromatin state even though a basal transcription of these regions has not only been detected but is now considered key to the maintenance of the condensed state, as it will be discussed later.

The vast majority of repetitive DNA consists of interspersed repeats, also called transposons or transposable elements (TEs) (Saleh et al., 2019). These have the capacity to migrate or replicate across the genome like many types of viral DNA sequences (Saleh et al., 2019). TEs are further classified into retrotransposons (comprising Long Interspersed Nuclear Elements (LINE), Short Interspersed Nuclear Elements (SINE) and long tandem repeats (LTR) retrotransposons, which include endogenous retroviruses) and DNA transposons (Chuong et al., 2017). Retrotransposons replicate via an RNA intermediate transcript which is later reverse transcribed into the target site (“copy and paste” mechanism). DNA transposons rely on a transposase to physically relocate to another site (Pace and Feschotte, 2007). The most abundant and important TEs in the human genome are the LINE L1 and the SINE *Alu*. Together they make up around 30% of the human genome. Most of these TEs are however silenced as a possible cellular mechanism of defense, but a relatively small number of them (labeled as retrotransposition-competent LINE-1 or RC-L1) is responsible for a large amount of nuclear transcription (Chuong et al., 2017; Saleh et al., 2019). Noticeably, while genomic repeats are, *per se*, silenced, a large fraction of TE fragments and low-complexity DNA is embedded and transcribed in mRNAs and ncRNA (Fort et al., 2021), and accounts for the largest share of nuclear transcription (Saleh et al., 2019).

Transposable elements were generally considered a remnant of a viral or parasitic insertion in the genome with no positive function and, if anything, a source of mutation and disease occasioned by their random insertion into promoters or coding sequences. Modern research has instead revealed that the repetitive RNA produced from TE may have been “exapted” or exploited by the cell to carry out some important functions in the regulation of gene expression (Chuong et al., 2017). For example, the *Alu* repeats were identified as a nuclear localization motif for RNA (Lubelsky and Ulitsky, 2018) and two thirds of lncRNAs are reported to contain TE elements (Chuong et al., 2017). Interestingly, the pattern of expression of long intergenic non-coding RNAs (lincRNA) shows a decrease in TE element content during differentiation, possibly highlighting a connection to cell stemness and early embryonal stages (Kelley and Rinn, 2012).

In the next paragraphs we will briefly examine the role of medium repetitive RNAs (such tandem and interspersed) in the establishment and maintenance of the two heterochromatin and the euchromatin states.

## Heterochromatin and Associated Repetitive RNAs

### Constitutive Heterochromatin

Constitutive Heterochromatin is the transcriptionally silent, permanently condensed form of chromatin (Janssen et al., 2018). It is a state set and maintained in regions such as the chromosome centromere and telomeres, which have important structural roles and encode no proteins. Both regions are characterized by tandem repeat DNA, although of a different kind.

Centromeres are the sites of kinetochore attachment during mitosis and have obvious critical importance in cell survival (Aldrup-Macdonald and Sullivan, 2014). They vary in sequence among species but in humans and primates they contain α-satellite DNA, a long tandem repeat sequence of a unit (the Higher Order Repeat, HOR), which is itself formed of a specific number of 171 bp monomers. The α-satellite DNA is surrounded by a pericentromeric region formed by the same monomers but without the HOR organization (Aldrup-Macdonald and Sullivan, 2014).

Centromeric HC is defined by the presence of H3K9 methylation (H3K9Me; Me1, Me2, Me3 : mono-, di- and tri- methylation), common to all constitutive HC forms. This marker is deposited by histone methyltransferase SUV39 and recognized by HP1 (Heterochromatin Protein 1); however, RNA, and specifically the repetitive RNA proceeding from the centromeric region, is necessary for histone methylation to take place and silencing to be maintained (Velazquez Camacho et al., 2017). In the fission yeast *S. pombe* the silencing occurs via an RNA-dependent RNA polymerase (RdRP) acting on transcribed repeat RNA and forming dsRNA which on one hand triggers the Argonaute protein binding and subsequent transcriptional silencing and, on the other hand, recruits an RNA-induced transcriptional silencing (RITS) complex able to bind the histone methyltransferase Clr4 (homolog of human SUV39) to deposit the required H3K9Me marks. A feed-forward mechanism sees these methylated H3 recognized by another protein (Sw16, homolog of human HP1) (Li and Fu, 2019).

While this mechanism has been very well studied in yeast, it has been ruled out in mammals by the lack of a mammalian RdRP (Li and Fu, 2019). An alternative mechanism suggested sees the intervention of piwi-associated RNAs (piRNA) in what has been labeled a “ping-pong” mechanism, which uses piRNA transcripts and retrotransposons to amplify the RITS/SUV39 methylation activity without the need for an RdRP (Li and Fu, 2019). It is also apparent that HP1 can directly bind centromeric repetitive RNA (Maison et al., 2011) just like SUV39H1 (Johnson et al., 2017). In summary, the stable silencing of centromeric chromatin—paradoxically—requires a certain baseline amount of centromeric transcription (and repetitive RNA molecules), in what is considered to be a mechanism designed to re-establish the silencing following DNA replication, which removes epigenetic marks and allows HC to be briefly transcribed (Volpe et al., 2002).

**FIGURE 1:**
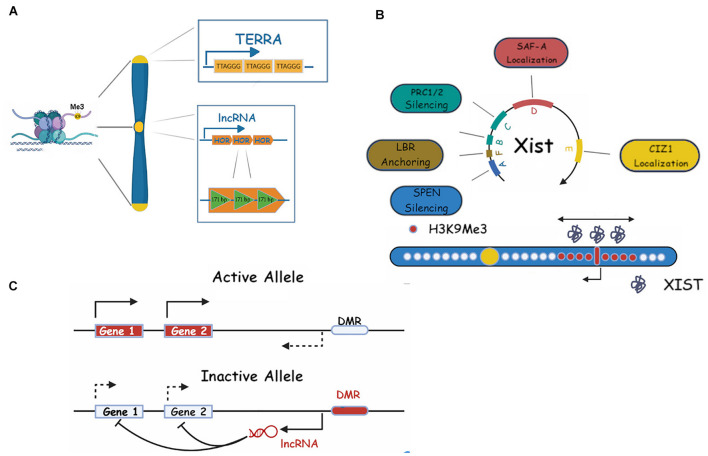
Repetitive RNAs in heterochromatin maintenance. **(A)** Centromeric (dark yellow) and telomeric (light yellow) constitutive heterochromatin and their associated ncRNAs (HOR and TERRA, respectively). ncRNA repeats (TERRA) or its length (HOR) are shown. **(B)** Xist RNA repeats and their roles in facultative heterochromatin establishment on the X chromosome are shown. Xist RNA is shown as gray molecules around the X inactivation center, H3K9me3 is shown as red dots, bidirectional arrows show the spreading in 2D, single pointed arrows indicate Xist transcription (see main text for more details). **(C)** A schematic representation of a classic/generic Imprinting Control Region (ICR) and its associated RNAs, in imprinting. Genes, DMR (differentially methylated regions) and lncRNAs are shown (see text for more details).

Telomeres are found at the terminal sequences of every eukaryotic chromosome and their main function is to preserve chromosome integrity during each round of DNA replication. Critically, they prevent the recognition of chromosome ends as DNA breaks and also limit somatic cellular proliferation by inducing senescence when shortened below a critical length (Palm and de Lange, 2008). Telomeres are constitutively silenced and contain the conserved tandem repeat TTAGGG along several kb (10–15 in humans, 20–50 in mouse). The actual chromosome end is arranged in a complex loop structure made possible by a 50–500 bp 3′ overhang of the + (“G”) strand (McElligott and Wellinger, 1997). The telomeric region is transcribed into a long non-coding RNA called TERRA (TElomeric Repeat containing RNA) whose promoter and transcription start lie in a poorly defined subtelomeric region containing a conserved 61 bp element, followed by 29 bp, and 37 bp repeats (61–29–37 repeats) (Cusanelli and Chartrand, 2014; Figure 1A). TERRA transcripts vary in length between a hundred bp and about 9 kb and stem from the − (“C”) telomeric strand, therefore being copies of the + strand and G-rich (Palm and de Lange, 2008). The role of TERRA in telomere maintenance is still the object of active research but it is clear that it is involved in telomere elongation via telomerase regulation and heterochromatin formation and maintenance. Indeed, TERRA has been shown to be bound by HP1 and to bind H3K9Me3 and its expression negatively correlates with this histone PTM. It also binds the Origin Replication Complex (ORC) and subunits of the Shelterin telomer complex (TRF1 and 2). Furthermore, siRNA depletion of TERRA results in H3K9 hypomethylation of the telomeric region (Deng et al., 2009), highlighting its role in stable telomere silencing.

### Facultative Heterochromatin

This term describes chromosomal regions which are silenced in a conditional way, being transcribed or silenced when circumstances, such as a certain stage of embryonic development or tissue differentiation, require it (Cerase et al., 2015; Żylicz and Heard, 2020). Facultative heterochromatin is also characterized by histone PTMs. For instance, during the process of X chromosome inactivation (XCI), the methylation of lysine 27 on Histone 3 (H3K27Me), deposited by the Polycomb repressive complex 2 (PRC2) is common in mouse, and H3K27me3 and H3K9me2–3 are instead common in humans (Chadwick and Willard, 2004; Schuettengruber et al., 2017). The typical fHC scenario is that in which a region of the genome becomes specifically silenced in a developmentally regulated process. In this case, the resulting heterochromatin can remain irreversibly condensed, be temporarily reactivated (Patrat et al., 2009) or even be silenced again (Cerase et al., 2015), via incompletely understood mechanisms.

In this review, we will focus our attention to two of the most studied fHC events: XCI and genomic imprinting (Żylicz and Heard, 2020).

*X chromosome inactivation* is a universal event in female mammalian development. As females have two X chromosomes, the simultaneous expression of both would result in abnormal protein dosage and lethal consequences (Borensztein et al., 2017). As a compensation mechanism, one of the X chromosomes is inactivated in the early stages of embryo development. Interestingly, in mouse, the event unfolds in two waves: in an early silencing step, at the four-cell stage, the paternal X chromosome is inactivated, after which reactivation occurs and at the implantation stage a second XCI wave, this time occurring on a random chromosome, takes place (Patrat et al., 2009). XCI is a complex process articulated in several stages, all orchestrated by the master regulator lncRNA *Xist (X inactive specific transcript)* (Brown et al., 1991). This transcript, around 15–17 kb long, contains six tandem repeats, labeled A to F, which are essential for Xist activities (Pintacuda et al., 2017b; Figure 1B). Repeat A consists of 8.5 copies (7.5 in mouse) of a 24 base pair sequence. It is necessary for XCI to occur and it has been shown to directly bind SPEN (SPlit ENnds homolog) (Monfort and Wutz, 2020). This binding recruits the histone deacetylase HDAC3 to deacetylate histone H3 thus clearing the way for repressive post-translational modifications (PMTs) (Monfort and Wutz, 2020). The Xist A repeat has been suggested, by modeling studies and experimental data (Lu et al., 2020a) to provide a scaffold for RNA-RNA interaction and hence multimerization of the transcript (Duszczyk et al., 2011). It is tempting to somehow link this multimerization process to the mechanism of the coating event, however, direct experimental evidence is currently not available. A recent paper by Rodermund et al. (2021) has shed light on the temporal mechanism of Xist spreading via time resolved structural illumination microscopy (3D-SIM). This study highlighted the role of the A repeat and related binding proteins such as Spen in mouse ES cells and neuronal progenitor cells (NPCs), in the spreading process. Repeat B, which consists of 32 copies of a cytidine rich hexamer, and C, which contains 14 copies of a 120-nucleotide unit, directly binding hnRNPK, recruit the Polycomb factor PRC1 which results in ubiquitination of H2AK119 (Pintacuda et al., 2017a). This modification is in turn recognized by the PRC2 complex which deposits the final H3K27 silencing modification (Blackledge et al., 2015). Repeat D contains multiple copies of a 290 bp unit and has been shown to bind the nuclear scaffold organizer SAF-A (Creamer and Lawrence, 2017) (3D-SIM), although SAF-A has been shown to bind Xist RNA broadly (Cirillo et al., 2016). Repeat E, a high number (50+) repetition of a 25 bp unit, binds the CIZ1 protein which does not appear to be essential for development (Khan et al., 2018). However, its deletion triggers a lymphocyte proliferative disorder in turn linked to X dosage compensation defects, and correspondingly a delocalization of Xist and partial derepression of Xi-silenced genes (Ridings-Figueroa et al., 2017), probably through its spreading role in differentiated cells (Pinter, 2016). The short repeat F is composed of two copies of a 10-mer repeat and is involved, together with repeat A/E, in the binding to the Lamin B Receptor (LBR), a mediator of the anchoring of the Xi chromosome to the heterochromatin rich nuclear periphery. Disruption of this bond results in Xi mislocalization and various degrees of silencing defects of the Xi genes (Chen et al., 2016). The spatial kinetics of XCI is also of great interest. While the end result is complete X inactivation via extensive chromosome coating by *Xist*, this process follows a spatial radiation from the *Xist* locus itself to the *Xist* entry site (XES) with genes in the proximity of these loci being silenced first. It is of note that the XES lies in 3D proximity to the *Xist* locus (Engreitz et al., 2013), suggesting a diffusion mediated mechanism for the *Xist* spread onto the X chromosome (Rodermund et al., 2021). A remarkable characteristic of XCI is its irreversibility following just a few days of differentiation. In fact, *Xist* is no longer required for long term maintenance of the Xi state after this period (Wutz and Jaenisch, 2000). It is believed that the state is maintained either by histone PTM (such as histone deacetylation, H3K27me3/H3K9me2–3) or by CpG island (CGI) promoter methylation (Żylicz and Heard, 2020), or stable protein accumulation (Cerase et al., 2019; Pandya-Jones et al., 2020) (discussed below).

The mechanism of Xist spreading and chromosome inactivation is not completely clear. It is known that the YY1, Ciz1 and SAF-A RNA binding proteins are required for Xist RNA spreading and silencing. Furthermore, the deletions of the Xist D repeats or the RGG (RNA binding) repeats of SAF-A, in some cell lines but not others, are sufficient to disrupt the bond (Kolpa et al., 2016). This has led to the suggestion that these repeat sequences act as multivalent scaffolds to promote multimerization of the RNA/protein complex (Pinter, 2016). There is conflicting data regarding the redundancy of the SAF-A RGG repeats and the indispensability of its DNA binding domain (Kolpa et al., 2016), therefore the matter is presently not yet settled. Noticeably, deletion of Ciz-1 also leads to loss of Xist localization and its diffusion in the nucleoplasm, a feature which can be rescued by the rexpression of Ciz-1 (Ridings-Figueroa et al., 2017). It is possible that Xist RNA uses different chromatin anchors in different tissues and/or developmental stages.

### Genomic Imprinting

Imprinting is the process by which some genes (in mammalian genomes thought to represent a small percentage) are exclusively expressed in a monoallelic fashion, i.e., by only one of the parental alleles, the other one being permanently silenced (Tucci et al., 2019; Figure 1C). One example is the human Igf2 gene, only the paternal copy of which is ever expressed (DeChiara et al., 1991). About 80 such genes have been identified so far and all their loci follow a similar expression pattern depending on the parent of origin (PoO): the region contains a demethylated CpG region called DMR (Differentially Methylated Region) also variously called IC (Imprinting Center), ICE (Imprinting Control Element), ICR (Imprinting Control Region) (Royo and Cavaillé, 2008).

These regions always express at least one lncRNA, whose expression PoO generally inversely correlates with the parent of origin of the expressed allele of the neighboring genes, i.e., expression of the paternal lncRNA will result in the expression of the maternal cluster protein coding genes (Royo and Cavaillé, 2008). The expression of the regulatory RNA is dependent on the methylation status of the DMR so that the methylated cluster will not express the ncRNA and will consequently express the protein coding genes. The direct mechanism for this activity has not been clarified but it is speculated that it may involve antisense binding (some lncRNAs are transcribed from the antisense strand of the protein gene) or small non-coding /microRNA activities. The end result is the *cis* silencing of the cluster protein coding genes of the PoO and consequent exclusive expression of the opposite parent’s.

A well-studied case is that of *kcnq1ot1*, a lncRNA located at the distal position of chromosome 7 (Mohammad et al., 2008). Its cluster controls a large number (at least eight) of genes that are always maternally expressed, *kcnq1ot1* being always expressed paternally. The lncRNA itself is long (90 kb) but truncation studies have shown that the minimal length required for silencing is 1.7 kb with a crucial internal 860 bp 3′ stretch (Mohammad et al., 2008). Interestingly, *Kcnq1ot1* contains five repeat sequences of 30 bp (MD1 repeats) which, however, do not seem to be strictly required for silencing (Mohammad et al., 2008). The silencing activity of *Kcnq1ot1* seems to be mediated, like that of *Xist*, by the deposition of H3K27Me3 PTM (Andresini et al., 2019), however, its action is limited to its genomic neighborhood.

## Euchromatin and Its Repetitive RNAs

**FIGURE 2:**
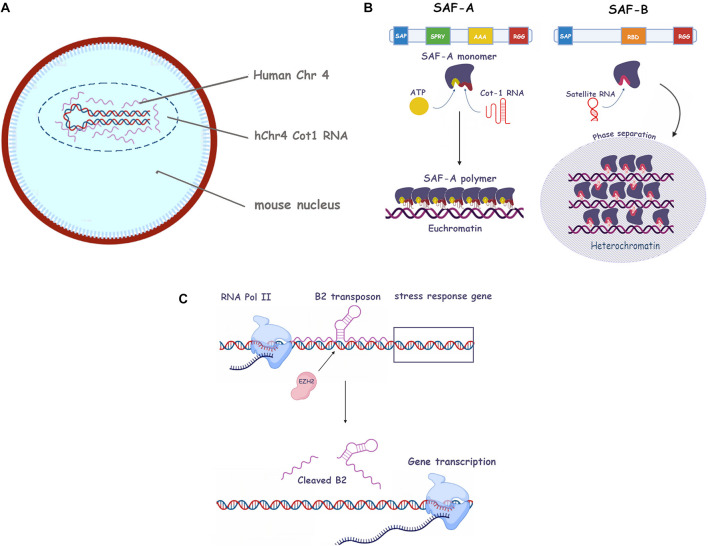
Roles of repetitive RNA in eu- and heterochromatin establishment and maintenance. **(A)** Human C0t-1 RNA specifically associates with the human chromosome in mouse nuclei containing a human chromosome (i.e. no association with mouse *in trans* RNA). **(B)** The SAF-A and SAF-B scaffold proteins and their associated repetitive RNAs. Protein domains such as SAP, SPRY, AAA, RGG, and RBD are shown (see text for more information). **Left**, SAF-A oligomerization in presence of ATP and C0t-1 RNA has been depicted (euchromatin). **Right**, SAF-B phase separation in presence of satellite ncRNA at centromeres (heterochromatin). **(C)** Mechanism of the B2 transposon mediated activation of stress response genes. RNA polymerase II (RNA Pol II), EZH2 stress response genes and B2 RNA are shown (see main text for more information).

Euchromatin, defined as the non-condensed, open, and transcriptionally active form of chromatin, lies at the opposite end of the spectrum to heterochromatin. As such, it is characterized by different histone PMTs and DNA methylation profiles which is quite distinct to that of HC, namely acetylation of histones or methylation on histone H3 Lys 4 and 36 among the others (Bannister and Kouzarides, 2011). However different the two chromatin states may be in appearance and behavior, they both share the feature of interacting with caRNA and, more specifically, with repetitive caRNA. A turning point in this research was the observation by Hall et al. (2014) that euchromatin is particularly enriched in C0T-1 repetitive RNA (Figure 2A). This RNA is the transcription product of the homonymous C0T-1 DNA, mostly known by researchers for its background suppression role in *in situ* DNA hybridization techniques and microarray screening. In fact, this DNA suppresses the spurious binding of ubiquitous repetitive sequences. Its name derives from a time parameter of re-hybridization experiments which originally led to its identification in the 70s (Holmes and Bonner, 1974). Hall’s remarkable findings imply that the nuclear RNA hybridizing to C0T-1 DNA is almost exclusively associated with euchromatin, that is species-specific (no cross reactivity between mouse and human euchromatin caRNA) and it “spreads” onto chromosomes much like Xist (Hall et al., 2014). This RNA appeared enriched in L1 and to a lesser extent, *Alu* repeats. The L1 component was revealed to be 5′ truncated, consistent with the knowledge that full-length L1 transposons are silenced in mammalian genomes (Rangwala et al., 2009).

The significance of finding a non-functional fragment of a transposable element as the most abundant component of euchromatin associated RNA is unclear (Hall et al., 2014). It is tempting to speculate that the 3′ L1 fragment retains the parent transposon’s capacity to somehow decondense DNA in order to effect its migration, but without the actual ability to integrate, and that this feature may have been exploited by the cell to maintain the euchromatin state (Jachowicz et al., 2017). In terms of mechanism, it has been shown that L1 RNA achieves its DNA decondensing effect by direct interaction with histone H2B, pointing at the electrostatics interactions of caRNA to histone binding as the rationale for the eu- to heterochromatin transition (Dueva et al., 2019).

## Mechanisms of Chromatin Regulation by Repetitive RNAs and RNA Binding Proteins

Having seen how caRNAs in general, and repetitive caRNAs in particular, play a decisive role in the regulation of chromatin states and, by extension, in gene expression, the question arises of what their mechanism of this action is. Given the high complexity of the nuclear microenvironment it can be predicted that the condensation state of chromatin will be a result of several interplaying components, mainly RNA and proteins, but also higher order structures such as Topological Associated Domains (TAD), Lamin Associated domains (LADs) (Collas et al., 2019) and phase separated spatial compartments within the nucleoplasm (Nozawa and Gilbert, 2019) (discussed below). In the following paragraphs, we will give a brief overview of the current state of research in the field, with a particular focus on critical selected examples of RNA binding proteins. For some extensive reviews on the roles of RNA binding proteins in nuclear and cellular functions, we refer the reader to the following articles (Díaz-Muñoz and Turner, 2018; Hentze et al., 2018; Gebauer et al., 2021).

### Scaffold Attachment Factors (SAF-A and B)

Two of the major protein partners of RNA in the regulation of chromatin state are the SAF (Scaffold Associated Factor) proteins, SAF-A and B (Nozawa et al., 2017; Figure 2B). SAF-A is a monomeric protein able to polymerize in an ATP-dependent fashion and to bind RNA through its RGG domain, in what appears to be a sequence independent way. Its association with RNA yields what has been defined as a “mesh” which contributes to maintaining the euchromatic state (Nozawa et al., 2017). Moreover, it can also bind A/T-rich double-stranded DNA sequences, known as scaffold attachment regions (SARs) (Xiao et al., 2012). This feature probably explains its ability to “bridge” the two nucleic acids to form the mesh. SAF-A (also known as HNRNPU) depletion in mouse hepatocytes results in an enhanced condensation and aberrant lamin association of DNA (Fan et al., 2018). The authors also observe its association with euchromatin. However, it would be inaccurate to draw a direct correlation between SAF-A and euchromatic DNA as the protein is also abundantly present on the inactivated X chromosome and a necessary partner of *Xist RNA* (McHugh et al., 2015). It appears that SAF-A is thus an RNA co-factor able to carry out different functions depending on the RNA (or RNA repeats) it associates with. Of note, the Xi associated SAF-A may be post translationally modified as it is not recognized by some antibodies (Nakagawa and Prasanth, 2011).

SAF-B is a cognate protein of SAF-A, sharing with it its SAP (serum amyloid P domain, a DNA-binding motive allowing direct binding to the matrix scaffold attachment regions) and RGG domains. It likewise binds RNA to modulate chromatin condensation. However, its role appears to be distinctly repressive (Huo et al., 2020; Figure 2B). It is able to bind repetitive pericentromeric ncRNA and its depletion causes the disappearance of H3K9Me3 foci, while at the same time leaving the total amount of H3K9Me3 unchanged. The authors hence suggest the protein may act as a trigger for phase separation, which we will cover later in our review.

### LncRNA Control of Nuclear Localization: Matrin 3 and CIZ1

The functions of proteins binding to the *Xist* E repeats have been recently studied in order to understand lncRNA spatial localization and function within the nucleus (Ridings-Figueroa et al., 2017; Pandya-Jones et al., 2020). Ridings-Figueroa et al. (2017) showed that CIZ1 interaction with Xist RNA allows proper anchoring of the Xi to the nuclear matrix thanks to its nuclear matrix bound C-terminus. Pandya-Jones et al. (2020) have identified several binding partners of Xist (Matrin 3, PTBP1, TDP43 and CELF1) and pinpointed their binding site to the E repeats, which would then act as a multimerization scaffold and as a seed for protein aggregation and condensation. Once formed, the protein condensate can then survive Xist removal and could explain the eventual dispensability of Xist in maintaining the Xi state (Csankovszki et al., 2001). Noticeably, Matrin 3 has also been associated to the nuclear retention and localization of Charme lncRNA, potentially suggesting a wider role of this protein in lncRNA/RNA nuclear retention (Desideri et al., 2020).

### RNA Degradation Machinery and Alu and B2 in Stress Response

The nucleus sees a constant turnover of RNA, mostly because of mRNA splicing and intron generation. Introns have a much shorter half-life than lncRNAs, and their degradation is mediated by ribonucleases such as XRN2 and the exosome complex (Nozawa and Gilbert, 2019). Other lncRNAs have a longer half-life of several hours, comparable to that of mRNAs, possibly because of their polyadenylation. The possible exceptions are the repetitive RNAs arising from constitutive heterochromatin (see above), whose transcription at a basal level is necessary to promote local HC condensation but whose overexpression is actually detrimental to the maintenance of the HC state, as shown in *Drosophila melanogaster* by Eberle et al. (2015). RNA degradation is therefore required at least for the heterochromatic silencing of the centromeric regions, which bind exosome subunits (Oya et al., 2013).

As another example highlighting the complexity of the repetitive RNA/chromatin relationship, we can quote the intriguing mechanism of the mammalian genetic stress response, as elucidated by Hernandez et al. (2020). To orchestrate the response to cellular stress, thermal or Estrogen Receptor (ER), a number of previously silenced genes are rapidly reactivated. The authors demonstrate that this silencing is actually mediated by B2 retrotransposons binding to target genes, and that the stress signal recruits EZH2, a subunit of the PRC2 complex, to the relevant loci (Figure 2C). This subunit promotes cleavage of the SINE B2 RNA, its release from chromatin and the transactivation of the stress genes. A similar behavior was observed with the other SINE, *Alu*. The phenomenon is interesting because it does not only shows a clear exaptation of the “parasitic” SINEs, but also uncovers a novel activity (RNAse enhancer) of EZH2, previously encountered as a histone methyltransferase. Moreover, in this context, its activity is not a transcriptional repressor but an activating protein (Hernandez et al., 2020).

### RNA-DNA Triplex

**FIGURE 3:**
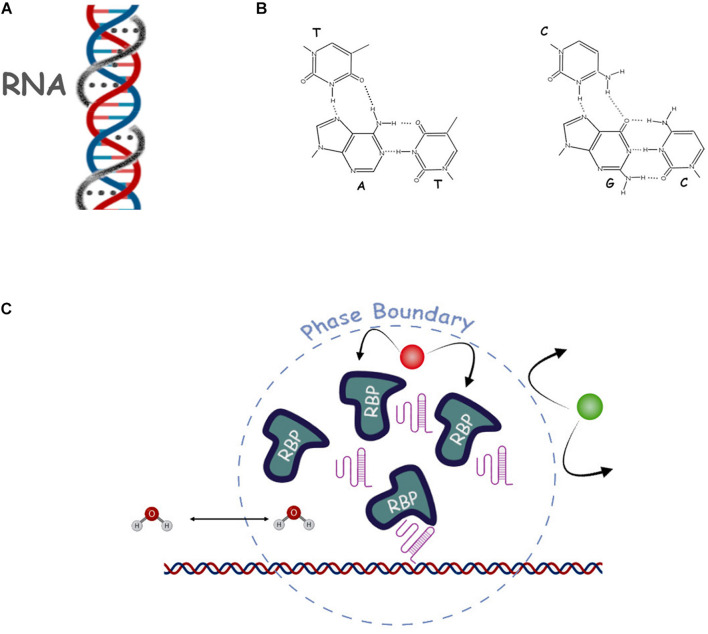
Selected proposed mechanisms of repetitive RNA action. **(A)** Scheme of the DNA/RNA triple helix. The third DNA strand and Hogsteen bonds are shown by the gray line and gray dots. **(B)** Hoogsteen triple base pairing contacts are shown in more detail (dashed lines). **(C)** Schematic of a nuclear phase separation where we show some of the main actors such as RNA (pink structured lines), RNA binding proteins (RBP), DNA (double helix), water (H2O) and a non-affine molecule (in green), and the phase-separated dynamic boundary (dashed line).

Soon after the classical DNA double helix structure was elucidated (Watson and Crick, 1953, 1974), speculation arose that it may not be the only possible stable structure and that, in particular, a triple helix with a single strand of DNA or RNA may be formed with the third strand inserting into the major groove and forming additional hydrogen bonds with DNA base pairs called Hoogsteen base pairs (Ghosal and Muniyappa, 2006). These triplex structures were eventually found *in vitro* (Morgan and Wells, 1968; Li et al., 2016) and later *in vivo* with RNA as the most stable third strand (Figures 3A,B). Moreover, the triplexes can have a silencing effect on transcription even in *trans*, possibly by recruiting PRC2, but also activating effects by binding p300/CBP (Li et al., 2016). The RNAs involved in these events are lncRNAs and particular repeating motifs (e.g., AG and TC) or palindromic sequences are thought to be needed to stabilize the interaction. The helix model would elegantly explain the role of some classes of lncRNAs, and especially the role of their sequence, in targeting the regions of DNA to activate or silence chromatin, however, the evidence in this regard is still preliminary (Ghosal and Muniyappa, 2006). One noticeable example, however, is the regulation of TGF-β pathway genes by the lncRNA MEG3 (Mondal et al., 2015), which is mediated by triple helix structures. By using CHiP with anti-triplex antibodies, the authors were able to determine enrichment of the triple helix structures at the TGFBR1, TGFB2 and SMAD2 genes which was lost upon removal of the MEG3 RNA.

## Repetitive RNAs in Nuclear Architecture

### Repetitive RNAs and Membrane-Less Organelles

One of the nucleus’ most remarkable features is the presence of membrane-less organelles, that are regions of distinct composition to the surrounding nucleoplasm but devoid of material barriers, such as phospholipid bilayers, to enforce the separation (Hyman et al., 2014). Examples are the nucleoli, the Cajal bodies, and paraspeckles. In this review, we will focus on the role of RNA, and in particular repetitive RNA in the formation of membrane-less nuclear compartments (Figure 3C).

It is thought that membrane-less compartments can exist thanks to a physico-chemical phenomenon known as phase separation, by which regions of a solution spontaneously split from the rest to form a distinct phase with different physical properties and chemical composition (Hyman et al., 2014). While such events are commonly observed in the presence of different solvents, they are a rarer occurrence in biological solutions (Titus et al., 2020). There are three main classes of phase separation: Liquid-Liquid (LLPS), Liquid-Gel (LGPT) and Polymer-Polymer (PPPT), depending on the type of solutes included in the different phases (Frank and Rippe, 2020). It is believed that the presence of high local concentrations of specific RNA, acting as a seed for condensation, and proteins can cause phase separation and the formation of membrane-less compartments. Different local chemical composition of these condensates would allow for differential chromatin transcriptional states and different rates of transcription, RNA processing and splicing (Huo et al., 2020). It is also thought that repetitive RNAs are key to the occurrence of this phenomenon in the nucleus, as their repeated motifs could work as multivalent ligands for RBP which in turn could result in a non-covalent RNA-protein “polymer” scaffold, initiating, either a LLPS or a LGPS process. The hallmarks of LLPS are: (i) a roughly spherical conformation (droplet); (ii) the ability to fuse and split (fusion and fission); (iii) concentration dependent aggregation and disaggregation and iv) the possibility of reversible disruption by specific chemicals (i.e., hexanediaol) (Sawyer et al., 2019).

The most studied membrane-less compartment, the nucleolus, is a large organelle that also has a role in genome organization and clusters heterochromatin at its periphery in the form of nucleolus-associated domains (NADs) in all somatic cells (Németh et al., 2010; van Koningsbruggen et al., 2010). NADs are, therefore, heterochromatic regions of low gene density and low gene expression (Vertii et al., 2019), and centromeres and telomeres often associate to nucleoli (Carvalho et al., 2001; Weierich et al., 2003; Zhang et al., 2004). For example, the inactive X chromosome was found to contact the perinucleolar compartment during mid/late-S-phase and it was suggested that this location could be important for faithful duplication of silent chromatin (Zhang et al., 2007). The nucleolus is formed by ribosomal DNA (rDNA) and RNA (rRNA) and RBPs (e.g., nucleophosmin, fibrillarin etc.). It collapses into an irregular structure upon inhibition of transcription or by depletion of RNA, suggesting that its integrity is not only dependent on the presence of RNA/repetitive RNA but also on RNA production (Boisvert et al., 2007).

Under heat shock and acidosis conditions, the expression of ribosomal intergenic spacer long non-coding RNA (IGS lncRNA) is induced and these transcripts are essential and sufficient for the immobilization of proteins that contain a nucleolar detention signal (NoDS) within the nucleolus. The mature IGS transcripts then tether the molecular complexes to their sites of expression on the rDNA cassette. These ncRNAs contribute to dissolving the characteristic tripartite organization of the nucleolus (Granular Compartment, Fibrillar Centers and Dense Fibrillar Components), giving place to the “protein detention center” (DC), which is spatially, dynamically, and biochemically distinct. Upon removal of the environmental stressor, the ncRNAs are repressed, the DC is dissolved, and tripartite nucleolar organization is re-established (Jacob et al., 2013). This form of nucleolar detention could also be considered a regulated posttranslational regulatory mechanism.

As mentioned before, the nucleolus loses its integrity when Pol II is inhibited, which is remarkable given the high rRNA content of the organelle. It is thought that Pol II provides stabilizing caRNA in the form of *Alu* repeats proceeding from intron splicing events in transcription hubs (Caudron-Herger et al., 2015). Transposon-associated ncRNAs represent one of the best examples of how transposable elements-derived ncRNAs (TE) modulate the spatial organization of nucleolus/genome. Alu RNA repeats interact with the nucleolin protein and thus contribute to the maintenance of nucleolar structure and function. Interestingly, Alu RNAs can target other genomic loci to the nucleolus suggesting that these ncRNAs may impact spatial genome organization by establishing physical links within and outside of the nucleolus (Caudron-Herger et al., 2015).

A recent study (Singh et al., 2018) shows how ncRNAs can help organize the nucleolus when associating with other forms of RNA and proteins. MiCEE (Mirlet7d, C1D, EXOSC10 EXOSC5 complex) was described as a ribonucleoprotein complex that mediates epigenetic silencing of bidirectionally expressed genes and is required for proper nucleolar organization. MiCEE acts by tethering the regulated genes to the perinucleolar region, inducing ncRNA degradation and transcriptional silencing. Specifically, the microRNA Mirlet7d forms a duplex with ncRNA/repeat-containing ncRNA expressed from bidirectionally transcribed genes and associates to C1D protein. C1D, in turn, targets the RNA exosome complex and the polycomb repressive complex 2 (PRC2) to the bidirectionally active loci. The exosome degrades the ncRNAs, whereas PRC2 induces heterochromatin and transcriptional silencing through EZH2 (Singh et al., 2018).

### Other Examples of Membrane-Less Sub-Nuclear Compartments

Early studies identified a role for molecular crowding in the formation of some membrane-less nuclear compartments (Richter et al., 2007; Cho and Kim, 2012), through liquid-liquid phase separation (Zhu and Brangwynne, 2015; Hall et al., 2019).

Nuclear RNAs and in particular, lncRNAs, have been shown to be involved in the formation of these sub-nuclear structures (Khosraviani et al., 2019). This is because lncRNAs exhibit properties (such as secondary structures) and repetitive elements that make them potential candidates for acting as architectural elements for chromatin organization and in this role are labeled architectural RNA (arcRNA). RNA forms secondary structures which interact with specific proteins and other RNA molecules. A single lncRNA can act as an RNA scaffold either by interacting with multiple copies of the same protein or several different proteins at once, representing the ideal seed molecule for condensation seeds. *Neat1* and *Malat1* and *Xist* (discussed above) are remarkable examples and are amongst the most conserved lncRNAs during vertebrate evolution although containing minimal repetitive elements.

The large isoform of *NEAT1* lncRNA (NEAT1.2) appears to play a critical role in organizing a type of nuclear compartment called paraspeckles, containing various mRNAs and RBPs. Targeted degradation of *NEAT1* disruptes the structure of these clusters (Chen and Carmichael, 2010). Moreover, many repeat-containing RNAs have been shown to associate with paraspeckles, suggesting that the domain might arise from clustering some specific classes of ncRNAs along with their RBPs (Prasanth et al., 2005). From a functional point of view, paraspeckles are dynamically “designed” to retain inside the nucleus certain mRNAs that had been subjected to high levels of adenosine-to-inosine editing and to concentrate certain RBPs to limit their functions in the nucleus.

*MALAT1* lncRNA localizes to compartments called nuclear speckles containing various splicing, RNA-processing and transcription factors, and that are thought to function as a storage for RNA-processing proteins when they are not actively engaged. Interestingly, *MALAT1* associates with actively transcribed genes in the periphery of nuclear speckles and dozens of RBPs, and these findings suggest that it could act as a scaffold mediating those interactions.

The pericentromeric-derived ncRNA, *HSATIII* arcRNA, leads to the formation of membrane-less nuclear compartments known as nuclear stress bodies. *HSATIII* arcRNA consists mainly of highly repetitive (GGAAU)_*n*_ sequences (Valgardsdottir et al., 2008) and is transcribed from the primate-specific pericentromeric satellite III regions under thermal stress conditions (Denegri et al., 2002; Jolly et al., 2004; Valgardsdottir et al., 2008). HSATIII arcRNAs remain stable in nuclei, but form membrane-less nuclear stress bodies (nSBs) upon recruitment of specific RNA-binding proteins such as Scaffold attachment factor B (SAFB), specific sets of SRSFs (SRSF1 and 9 during thermal stress), transcription factors HSF1 and CREBBP, bromodomain protein BRD4 (Jolly et al., 2004; Metz et al., 2004; Kawaguchi et al., 2015; Hussong et al., 2017) and many nuclear RBPs involved in pre-mRNA splicing and processing (Ninomiya et al., 2020). Similarly, *Hsr omega* (heat-shock RNA-omega) arcRNA from *Drosophila melanogaster* contains tandem repeats of 280 nt in a stretch of ∼10 kb that contribute to the recruitment of various RNA-binding proteins to omega speckles (thermal stress-induced nuclear bodies) (Prasanth et al., 2005; Singh and Lakhotia, 2015).

An excellent recent review summarizing the current knowledge about cell organization and membrane-less compartments was published recently by Quinodoz and Guttman (2021). We refer the author to this review for more details on these topics.

### Repetitive RNA in the Formation and Maintenance of Nuclear Domains

It has been suggested that nuclear RNAs are an essential component of interphase chromosomes (Hall and LawrencE, 2016) but it remains unclear which of these may have roles in shaping large-scale chromatin structure and regulating genome function. An example suggesting RNA−based roles in nuclear architecture is how the digestion of RNA, but not of proteins, resulted in a highly disorganized nucleus, as assessed by electron microscopy, and also mislocalization of chromatin regulatory complexes (Davidson and Britten, 1979; Nickerson et al., 1997; Bernstein et al., 2006; Britten, 2010). Also, the removal of RNA leads to the collapse of nuclear bodies providing clear evidence for the architectural role of RNA in the formation of these structures (Bond and Fox, 2009; Mao et al., 2011; Shevtsov and Dundr, 2011).

Regarding spatial genome organization, non-coding RNAs (ncRNAs) have particularly emerged as major regulators and can regulate transcription at the same locus (*in cis*) from where they are transcribed or elsewhere in the genome (*in trans*) (Khosraviani et al., 2019). Collectively, ncRNAs can impact genome organization by modulating perinuclear chromosome tethering, the formation of major nuclear compartments, chromatin looping, and various other chromosomal structures (Khosraviani et al., 2019). Remarkably, several ncRNAs from repetitive DNA loci have emerged as major players that mediate crosstalk between spatial genome organization, expression, and stability (Caudron-Herger et al., 2015). Considering that highly repetitive sequences (i.e., fragments of TEs) are found in most transcripts (including unspliced mRNA) and that they comprise up to 2/3 of the human genome, they might contribute significantly to the abovementioned nuclear roles. We also refer the reader to other reviews on ncRNAs, and particularly repetitive nuclear ncRNAs, mostly emerging from rDNA repeats, telomeric regions, transposable elements, and centromeres (Hall and LawrencE, 2016; van Steensel and Furlong, 2019).

### Role of ncRNAs in Lamin Associated Domains Generation and Maintenance

**FIGURE 4:**
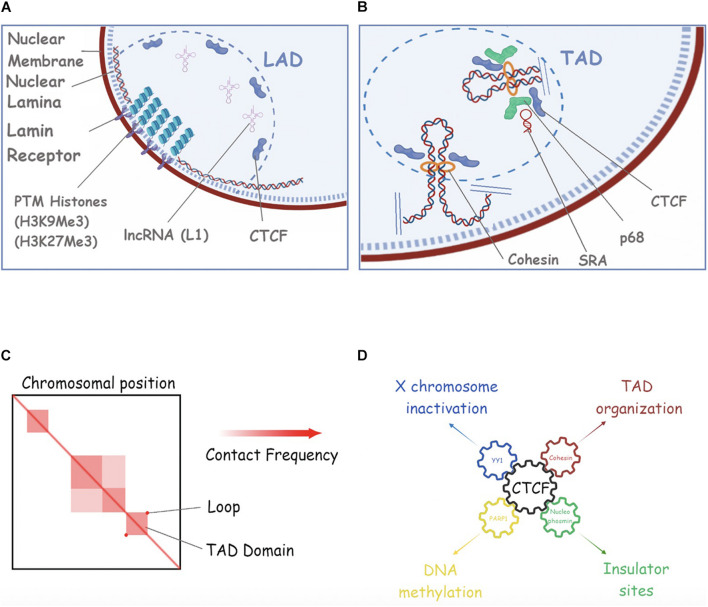
Repetitive RNAs in nuclear compartments. **(A)** A schematic of Lamina Associated Domains (LAD). In this picture we show the nuclear membrane (purple), the nuclear lamina mash (dotted blue), Lamin B receptor (Lbr) (light violet), Post-translational histone modifications (PMTs) as compacted histones (piled circles), L1 RNA as structured RNA filament, and CTCF (violet). **(B)** An example of Topology Associated Domain (TAD) in the nuclear space is shown. Cohesin, SRA, p68 and CTCF are shown (see text for more details). **(C)** An example of HiC analysis of 3D chromosomal domains (adapted from Rao et al., 2014) are shown. Compartments, subcompartments and loop/domains are shown. **(D)** CTCF is a key interacting player for the regulation of many cell processes such as X chromosome inactivation (through YY1), DNA methylation (though PARP1), TAD organization (through CTCF) and Insulation of DNA regions (through Nucleophosmin). See the main text for more details and references.

In mammalian cells, the nuclear lamina is thought to be the key organizer of the radial arrangement of chromatin in interphase nuclei, by creating a large nuclear compartment where most of the inactive chromatin clusters in the form of lamina-associated domains (LADs) (Peric-Hupkes et al., 2010; Kind et al., 2015; Figure 4A). LADs are typically 0.1–10 megabases, gene-poor, enriched in heterochromatin, and display low gene activity (Guelen et al., 2008; Lund et al., 2014; Khosraviani et al., 2019). The association of chromatin with the nuclear lamina through LADs aids functional organization of the genome and enables a spatio-temporal regulation of replication and transcription (Buchwalter et al., 2019).

Several studies might suggest that ncRNAs and especially, repetitive ncRNAs play a critical role in anchoring specific loci to the nuclear lamina, organizing genome architecture and regulating gene expression. *Xist* lncRNA is the clearest example, and its interaction with lamin B receptor (LBR) is required for tethering the entire X-chromosome to the nuclear lamina during XCI (Chen et al., 2017; Young et al., 2021). Apart from *Xist* RNA, there are a few examples of ncRNAs possibly regulating the localization of specific genetic loci to the nuclear lamina, and hence affecting gene expression. As an example, the *L1 repeat* RNA plays a role in sequestering L1-rich sequences and associates genes in inactive domains for silencing. Depletion of *L1* RNA in embryonic stem cells (ESCs) leads to the relocation of L1-enriched chromosomal segments from inactive domains to the nuclear interior and derepression of L1-associated genes. Moreover, *L1* RNA seems to facilitate the interaction of L1 DNA to proteins such as nucleolin to target it to NADs/LADs in mouse ESCs (Lu et al., 2020b).

Recent studies have highlighted the role of telomeric-derived ncRNAs in the crosstalk between telomeric heterochromatin and the subnuclear positioning of telomeres. This is the case for PIWI-interacting RNAs (piRNAs), which are typically transcribed from telomeric regions and play a pivotal role in the establishment and maintenance of telomeric chromatin in the germline, facilitating loading of HP1 and H3K9me3 at different telomeric regions. In the fly germline, the loss of some piRNAs decreased perinuclear telomere positioning and lowered the local enrichment of HP1 and H3K9me3, resulting in telomere dysfunction (Radion et al., 2018).

Similarly, transposable elements (TEs)-derived ncRNAs might play a role in chromatin compartmentalization. Enhancer lncRNAs (elncRNAs), often originating from repetitive sequences/fragments derived from transposable elements (Rebollo et al., 2012; Su et al., 2014) may reinforce the interactions between enhancers and promoters (Hou et al., 2019) as reported during developmental progression and tumor suppression. For example, in developing T cells, *ThymoD* ncRNA transcription promoted demethylation at CTCF bound sites and activated Cohesin-dependent looping to reposition the Bcl11b enhancer from the lamina to the nuclear interior and to juxtapose the Bcl11b enhancer and promoter into a single-loop domain. As expected, these large-scale changes in nuclear architecture were associated with the deposition of activating epigenetic marks across the loop domain (Isoda et al., 2017).

Sophisticated techniques are being developed to analyze nuclear DNA architecture with increasing accuracy and minimal interference. For example, in 2020, Girelli et al. (2020) developed a method called GPseq that allowed generating the first high-resolution map of radial chromatin organization in human cells. To infer radial locations throughout the genome, GPseq is used in combination with a special FISH assay called YFISH, which allows monitoring the pattern of *in situ* digestion before sequencing the GPSeq sample. Specifically, YFISH uses a Y-shaped adapter that will ligate the cuts introduced *in situ* by a restriction enzyme, and then the use of complementary fluorescently labeled oligos will help with the detection. In 2016 Brant et al. (2016) developed i3C as a chromosome conformation capture technique aimed at minimizing the *in vivo* interference of formaldehyde crosslinking (Brant et al., 2016). In order to stimulate future work in this direction, we suggest the use of these or similar techniques, in conjunction with existing LAD mapping techniques at population or single cell level (Kind et al., 2015) in different conditions to investigate the contribution of repetitive RNAs to LADs formation. For example, comparing the chromatin distribution in the presence and in the absence of a specific ncRNA or a specific class of ncRNA.

### Role of ncRNAs in Topologically Associated Domains Generation and Maintenance

At a large scale, chromosomes segregate into regions that form two different types of chromatin, referred to as “A-type” (euchromatin) and “B-type” (heterochromatin) genomic compartments (Lieberman-Aiden et al., 2009). At the sub-megabase scale, chromosomes form a series of architectural chromatin units termed topologically associated domains (TADs), each of which includes hundreds of kilobases of DNA (Dekker and Heard, 2015; Figures 4B,C). TADs are separated by regions known as TAD boundaries and exhibit a higher frequency of intradomain interactions compared to interdomain interactions (Rao et al., 2014; Dixon et al., 2015). The organization of the genome into TADs is critical for coordinated transcriptional regulation, chromatin states, and DNA replication. These structures dynamically change during differentiation (Dixon et al., 2015) and are perturbed in disease (Barutcu et al., 2015). Over the years, multiple studies have described the role of different types of ncRNAs in TADs formation and maintenance (Fudenberg et al., 2017; Amaral et al., 2018). Generally, the mammalian genome is arranged into compartments of active and inactive chromatin (Lieberman-Aiden et al., 2009). Moreover, linearly non-contiguous TADs can contact each other, defining long-range interactions that can vary between cell types and during differentiation (Szabo et al., 2018; Connelly et al., 2019; Paulsen et al., 2019).

Traditionally, Cohesin, loop extrusion, and CCCTC-binding factor (CTCF) have been proposed to create TADs to regulate gene expression. TADs boundaries are enriched for the insulator binding protein CTCF, housekeeping genes, transfer RNAs, and short interspersed element (SINE) retrotransposons, indicating that these factors may have a role in establishing the topological domain structure of the genome (Liyakat Ali et al., 2021). Cohesin can utilize its ATPase activity to extrude loops of chromatin and this is limited by CTCF-occupied insulator DNA elements (Sanborn et al., 2015; Fudenberg et al., 2017). This process is thought to structure and insulate chromosomes, limiting the effects of distal gene regulatory elements to genes within a given TAD. Importantly, altering TAD boundaries can lead to ectopic contacts between cis-regulating elements and gene promoters, and thus gene misexpression, which can contribute to developmental defects and cancer (Akdemir et al., 2020). However, there is recent evidence suggesting that the weakening of TAD boundaries also appears to be independent of CTCF binding and, transcription would have supporting roles in the formation of TADs and regulation of *inter−TAD* interactions (Barutcu et al., 2019). In this study, Barutcu et al. (2019) showed that the strength of TAD boundaries, measured by the degree of interactions that occur across a TAD boundary, is significantly decreased upon transcriptional inhibition, suggesting a role for total, steady−state single−stranded RNA on genome architecture. This finding is consistent with others where transcriptional inhibition, as well as transcriptional elongation can displace Cohesin from CTCF sites and disrupt chromatin interactions (Li et al., 2015; Heinz et al., 2018; Vian et al., 2018; Rowley et al., 2019), a phenomenon that correlates with the weakening of TAD boundaries. This evidence do not, however, exclude other possibilities (i.e., transcription inhibition weakens transcriptional condensates). Another supporting fact for the potential role of active transcription in the topological organization of the genome is the enrichment of transcription-associated RNAs with TAD boundaries (Bell et al., 2018) as well as the observation that active transcription is a stronger predictor for TAD partitioning in flies (Ulianov et al., 2016; Hou et al., 2019) than CTCF and Cohesin accumulation, the prototypical TAD boundary markers in mice and humans (Merkenschlager and Nora, 2016). As already speculated, pre-existing and newly transcribed RNA (which contains repeats) might play a role in genomic compartmentalization (Erdel and Rippe, 2018) and as Erdel and Rippe described, after RNase A treatment and before cross-linking, there is a subtle perturbation of compartmental interactions, especially in B−type compartments (Erdel and Rippe, 2018).

There are also a few studies supporting the role of ncRNAs in facilitating long-distance chromatin interactions through Cohesin-binding. In mammalian cells, for instance, not all the CTCF sites are co-occupied by Cohesin, suggesting that additional factors could dictate Cohesin binding at CTCF sites (Zlatanova and Caiafa, 2009). In fact, several partners of CTCF have been identified (e.g., YY1, Nucleophosmin, PARP), each associated with a particular and distinct function of the protein (Figure 4D). The interaction between Cohesin and CTCF is modulated by the DEAD-box RNA binding protein p68, together with its associated ncRNA called steroid receptor RNA activator (SRA), and promotes insulator function, for example, at the Igf2/H19 locus. Additionally, Cohesin was reported to bind to the ncRNAs transcribed on enhancer regions, termed enhancer RNAs (eRNAs) (Racko et al., 2018). For instance, the eRNAs bind to Cohesin and increase its recruitment to the enhancer regions in response to the ER ligand estradiol, stimulating the enhancer-promoter interactions in MCF7 breast cancer cells. Furthermore, key components of the Cohesin complex, SA1 and SA2, bind to various RNA containing substrates, including ssRNA, dsRNA, RNA: DNA hybrids and R-loops and it has been shown that both SA1 and SA2 Cohesin subunits localize to regions on dsDNA that contain RNA (Pan et al., 2020). Another example in this category is the blncRNA1 (boundary associated long non-coding RNA-1), generated from the CBS5 boundary element (HOXA locus) promoter activity. CBS5 employs both Cohesin and blncRNA1 to establish and maintain TADs at the HOXA locus and the transcript promotes proper expression of HOXA genes (Nwigwe et al., 2015).

Likewise, there are examples of ncRNAs facilitating long-distance chromatin interactions through CTCF binding. CTCF is found to interact with a multitude of transcripts genome-wide, both protein-coding mRNA and non-coding transcript, mRNAs as well as many long-non-coding RNA (lncRNA) such as well-characterized species from imprinted loci and previously unannotated transcripts from intergenic space (Kung et al., 2015). CTCF is recruited in a locus-specific manner and implicates CTCF-RNA interactions in long-range chromosomal interactions. For example, Tsix and Xist RNAs target CTCF to the X-inactivation centre, thereby facilitating homologous X-chromosome pairing (Kung et al., 2015). In accordance with this, it is not surprising that mutation of the RNA-binding regions in CTCF (ZF1 and ZF10) disrupts gene expression, chromatin binding, and the formation of chromatin loops (Saldaña-Meyer et al., 2019). Moreover, transcription inhibition disrupts CTCF binding to chromatin (Saldaña-Meyer et al., 2019). Interestingly, there are positionally conserved RNAs linked to chromatin organization structures called topological anchor point RNAs (tapRNAs). These tapRNAs overlap binding sites for the CTCF chromatin organizer and localize at chromatin loop anchor points and borders of TADs. Characterization of these ncRNAs and their associated coding genes shows that they regulate each other’s expression and influence the metastatic phenotype of cancer cells *in vitro* in a similar fashion (Amaral et al., 2018). Remarkably, and in relation to CTCF, a very recent study based on the CTCF CUT and RUN technique revealed that intact RNA, of unknown nature, is required for maintaining the chromatin environment around CTCF likely by facilitating local chromatin compaction (Thakur et al., 2019).

TEs-derived ncRNAs have been frequently described as involved in the establishment and maintenance of insulator boundaries between TADs. Transposable elements (TEs) are responsible for genomic instability, epigenetic silencing and are intrinsically linked to 3D organization as several studies described how they shape genome organization from demarcating TAD boundaries to harboring binding sites for architectural proteins (Diehl et al., 2020). Mammalian-wide interspersed repeats (MIRs) are a conserved family of TEs that have a substantial regulatory capacity and share sequence characteristics with tRNA-related insulators. MIR insulators appear to be CTCF independent and show a distinct local chromatin environment with marked peaks for RNA Pol III and several histone modifications. This suggests that MIR insulators recruit transcriptional complexes and chromatin modifying enzymes *in situ* to help establish chromatin and regulatory domains in the human genome (Wang et al., 2015). The primate-specific endogenous retrotransposon human endogenous retrovirus subfamily H (HERV-H) RNA represents another example and has a role in creating TADs in hPSCs and PSCs from other species and this ability depends on abundant transcription, as transcriptional repression of HERV-H elements prevents the formation of boundaries (Zhang et al., 2019). The Murine Endogenous Retroviral Element (MuERV-L/MERVL) family of transposable elements drives the 3D reorganization of the genome in the early mouse embryo by promoting the formation of insulating domain boundaries throughout the genome. The formation of these boundaries is coupled to the upregulation of directional transcription from MERVL, which results in the activation of a subset of the gene expression program of the 2-cell stage embryo (Kruse et al., 2019).

In spite of the previously mentioned findings, recent studies suggest that the pool of RNA in the cell appears to be largely dispensable for the maintenance of TADs (Fudenberg et al., 2017; Tan et al., 2017; Amaral et al., 2018). As the authors stated, while pre−existing transcribed RNA may play a role at small local scales or mediate inter−chromosomal interactions (Maass et al., 2012; Hacisuleyman et al., 2014), overall it does not appear to significantly influence TAD boundary formation. The fact that TAD boundaries remain intact in cells treated with RNase A, either before or after formaldehyde crosslinking, is consistent with a model explaining that TAD formation is primarily driven by DNA–protein and protein–protein interactions rather than by RNA (Fudenberg et al., 2017). Considering these contradictory results, more studies and accurate techniques need to be developed, in order to discriminate direct vs. secondary effects of RNA depletion.

### Repetitive RNAs Involved in Trans-Chromosomal Interactions

**FIGURE 5:**
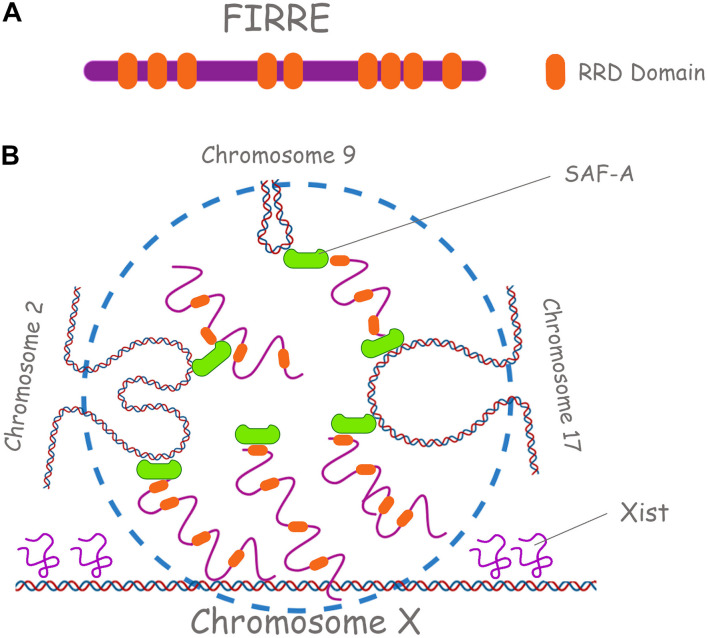
Repetitive RNAs underpin long range chromosomal interactions. **(A)** Firre lncRNA is shown, with its RRD domains (Repeating RNA Domain) in orange. **(B)** Firre has a role in the X chromosome inactivation (XCI) and in the localization of murine chromosomes 2, 7, and 17. SAF-A in green is also shown.

Linearly non-contiguous TADs can also contact each other, defining long-range interactions that can vary between cell types and during differentiation (Quinodoz et al., 2018; Szabo et al., 2018; Paulsen et al., 2019). For instance, repetitive and repeat-containing lncRNAs such as *XIST* and *FIRRE (functional intergenic repeating RNA element)*, colocalize with Xi and determine trans-chromosomal interactions (Figure 5). It is suggested that during XCI, *Xist* would facilitate the atypical TAD structure of the Xi into two “mega-domains” around the DZX4 locus (Bonora et al., 2018). These mega-domains, differently from those on the active X chromosome (Xa), exhibit random/semi-random pattern of interactions compared to the punctate interactions between specific loci seen on most chromosomes. With regards to *FIRRE lncRNA*, it interacts with SAF-A through its RRD (Repeating RNA Domain), a 156-bp repeating sequence (Hacisuleyman et al., 2014), contributing to the organization of higher-order chromosome architecture to spatially coordinate the regulation of genes involved in the same biological process (e.g., adipogenesis). This conserved and unique motif is necessary to localize *FIRRE* around its site of transcription in the nucleus but it can also localize any RNA containing it (Hacisuleyman, 2015). In mouse, *FIRRE* forms a punctate compartment in the nucleus where its locus on the X chromosome and several specific loci on mouse chromosomes 2, 9, 15, and 17 colocalize with it. *FIRRE* is then required for these inter-chromosomal interactions. Both genetic deletion of the Firre locus and knockdown of SAF-A resulted in loss of colocalization of these trans-chromosomal interacting loci (Hacisuleyman et al., 2014).

Similarly, TEs may be involved in the establishment of conserved long-range chromosomal interactions in different organisms and some of these interactions appear to be important in gene regulation. For example, in the fungus *Epichloe festucae*, these repeat-rich blocks mediate genome folding within the nucleus and help to divide the genome into distinct regions that have similar gene expression profiles, modulating in this way the expression of genes that are strongly differentially expressed (Winter et al., 2018).

Within the nuclear matrix, novel classes of repetitive RNAs have been identified as major players of the nuclear architecture and associated genome regulation. A class of repeat-containing lncRNA from the AAGAG satellite DNA repeat have emerged as a crucial component of the nuclear architecture in *Drosophila melanogaster* (Lohe and Brutlag, 1986; Lohe et al., 1993; Smith et al., 2007). Reduction of these repetitive RNAs results in disruption of the nucleoskeleton and, consequently, the assembly and stability of the chromosome compartments are disturbed (Pathak et al., 2013). In 2010, Zheng et al. (2010) identified a heterogenous population of GAA-repeat-containing RNAs (GRC RNAs) that primarily consist of polypurine repeats, ranging from 1.5 to 4 kb. These RNAs are distributed throughout the nucleus in a micropunctate pattern in both primary and transformed human and mouse cell lines. GRC-RNAs associate with the nuclear matrix and interact with several *bona fide* nuclear matrix proteins and have been proposed to play important structural roles in the maintenance of the nuclear and nuclear architecture and regulate gene expression.

## Final Remarks

Early in the human genome project it became clear that most our DNA is made of non-coding, repetitive sequences. Lacking any obvious function and carrying no protein coding information, these vast stretches of the genome were soon labeled “junk DNA” and were thought to be remnants of viruses that had lost replicative competence and merely multiplied inside our DNA, or even aberrant products of DNA replication errors.

It is now becoming more and more clear instead that, far from being genetic “deadwood” these repetitive expanses are actively and deliberately transcribed into non-coding RNAs which play a major role in regulating gene expression and silencing, organizing nuclear architecture, compartmentalizing the nucleus, and modulating protein function. We can now state with confidence that the study of repetitive RNA role and mechanism of action will open a new frontier in cell biology. We hope that our review will further stimulate research in the consolidating field of repetitive RNA biology.

## Author Contributions

AC conceived the idea of this review. AC, NB, and GT wrote the manuscript and selected representative examples. All authors contributed to the article and approved the submitted version.

## Conflict of Interest

The authors declare that the research was conducted in the absence of any commercial or financial relationships that could be construed as a potential conflict of interest.

## Publisher’s Note

All claims expressed in this article are solely those of the authors and do not necessarily represent those of their affiliated organizations, or those of the publisher, the editors and the reviewers. Any product that may be evaluated in this article, or claim that may be made by its manufacturer, is not guaranteed or endorsed by the publisher.
